# Targeting Small Molecule Delivery to the Brain and Spinal Cord via Intranasal Administration of Rabies Virus Glycoprotein (RVG29)-Modified PLGA Nanoparticles

**DOI:** 10.3390/pharmaceutics12020093

**Published:** 2020-01-24

**Authors:** Eugene P. Chung, Jennifer D. Cotter, Alesia V. Prakapenka, Rebecca L. Cook, Danielle M. DiPerna, Rachael W. Sirianni

**Affiliations:** 1 Barrow Brain Tumor Research Center, Barrow Neurological Institute, Phoenix, AZ 85013, USA; epchung25@gmail.com (E.P.C.); aprakapenka@gmail.com (A.V.P.); rebeccalmccall@gmail.com (R.L.C.); dannidiperna@gmail.com (D.M.D.); 2 School of Life Sciences, Arizona State University, Tempe, AZ 85281, USA; 3 Vivian L. Smith Department of Neurosurgery, McGovern Medical School, Houston, TX 77030, USA; Jennifer.D.Cotter@uth.tmc.edu

**Keywords:** intranasal, nanoparticle, brain, spinal cord, targeting, rabies virus glycoprotein

## Abstract

Alternative routes of administration are one approach that could be used to bypass the blood–brain barrier (BBB) for effective drug delivery to the central nervous system (CNS). Here, we focused on intranasal delivery of polymer nanoparticles. We hypothesized that surface modification of poly(lactic-*co*-glycolic acid) (PLGA) nanoparticles with rabies virus glycoprotein (RVG29) would increase residence time and exposure of encapsulated payload to the CNS compared to non-targeted nanoparticles. Delivery kinetics and biodistribution were analyzed by administering nanoparticles loaded with the carbocyanine dye 1,1′-Dioctadecyl-3,3,3′,3′-Tetramethylindotricarbocyanine Iodide (DiR) to healthy mice. Intranasal administration yielded minimal exposure of nanoparticle payload to most peripheral organs and rapid, effective delivery to whole brain. Regional analysis of payload delivery within the CNS revealed higher delivery to tissues closest to the trigeminal nerve, including the olfactory bulb, striatum, midbrain, brainstem, and cervical spinal cord. RVG29 surface modifications presented modest targeting benefits to the striatum, midbrain, and brainstem 2 h after administration, although targeting was not observed 30 min or 6 h after administration. Payload delivery to the trigeminal nerve was 3.5× higher for targeted nanoparticles compared to control nanoparticles 2 h after nanoparticle administration. These data support a nose-to-brain mechanism of drug delivery that closely implicates the trigeminal nerve for payload delivery from nanoparticles via transport of intact nanoparticles and eventual diffusion of payload. Olfactory and CSF routes are also observed to play a role. These data advance the utility of targeted nanoparticles for nose-to-brain drug delivery of lipophilic payloads and provide mechanistic insight to engineer effective delivery vectors to treat disease in the CNS.

## 1. Introduction

Delivery of therapeutic molecules to the central nervous system (CNS) is severely limited by the blood–brain and blood–spinal cord barriers (BBB and BSCB). These barriers are comprised of tight junctions and efflux pumps that restrict parenchymal penetration of all but a fraction of small, lipophilic molecules. The intranasal route has been proposed to be an alternative to parenteral delivery that could bypass the BBB to enable more direct delivery of molecules to the brain. Many different molecules have been successfully delivered to the CNS through the intranasal route, including proteins, polynucleotides, and small molecule drugs [1,2,3,4,5,6,7]. Intranasal administration has been shown to have several key advantages over systemic administration, including avoidance of first-pass metabolism and ease of administration [8]. This can enable better drug delivery to the brain, since substances have the potential for more direct access to the CNS than if they had been administered by routes that encounter first-pass metabolism. Several direct nose-to-brain transport routes that bypass the BBB have been proposed [9]. Unsurprisingly, a variety of intranasal therapeutics have been clinically tested or approved for use in humans [10].

Although intranasal drug delivery has been a useful approach for CNS drug delivery, freely administered agents can still encounter pharmacokinetic limitations. Freely administered molecules can sometimes be poorly bioavailable or are cleared rapidly with draining mucous [10]. One solution to this issue is the employment of nanoparticle drug delivery systems. Polymeric nanoparticles are a popular material choice for drug delivery and have been shown to enhance CNS delivery of a drug relative to freely solubilized drug provided by the intranasal route [11,12]. To further enhance brain-specific delivery and reduce off target exposure, several groups have surface-modified nanoparticles with targeting ligands, utilizing receptor mediated transporters to increase CNS-specific delivery of nanoparticles or their encapsulated payloads [13].

To develop a brain-targeted nanoparticle system for intranasal administration, we focused on a 29 amino acid peptide component of rabies virus glycoprotein (RVG29). RVG29 is derived from the rabies virus coat protein, and it is both necessary and sufficient for viral neurotropism [14]. It is thus a promising new candidate for CNS targeted drug delivery. We previously showed that surface modification of nanoparticles with RVG29 transiently enhances payload delivery to the brain when nanoparticles are administered intravenously [15]. In this prior work, we observed substantial differences in delivery and targeting by specific tissue region within both the brain and spinal cord. These data yielded evidence that, in contrast to motor neuron-mediated uptake of native virus, RVG29 facilitated CNS drug delivery via interactions with gabba-aminobutyric acid B (GABA_B_) receptors that are non-uniformly expressed across the CNS.

Given that RVG29 receptors are also expressed highly on the trigeminal nerve [16,17,18], which is known to be a route of direct transport from the nose to the brain [19], we hypothesized that surface modification of nanoparticles with RVG29 could increase payload exposure to the CNS following intranasal administration. To test this hypothesis, we used nanoparticles composed of poly(lactic-*co*-glycolic acid) (PLGA), encapsulating the lipophilic carbocyanine dye DiR, and surface modified via avidin-biotin interactions to display RVG29. Non-targeted (ctr-NPs) and targeted (RVG-NPs) nanoparticles were administered to mice via the intranasal route, and the brain, spinal cord, and peripheral tissues were extracted at multiple time points for biodistribution analysis. The brain and spinal cord were dissected into major anatomical regions to gain insight into potential mechanisms of payload distribution in the CNS. These intranasal data were compared to equivalent data previously collected for intravenous administration [15]. Our results demonstrate that surface modification of nanoparticles with RVG29 can achieve to specific tissues within the CNS following intranasal administration by mechanisms that are distinct from intravenously administered nanoparticles. We observe a central role in CNS drug delivery for nanoparticle interactions with the trigeminal nerve following intranasal administration. Taken in sum, these studies advance RVG29-modified nanoparticles as a candidate for intranasal administration of small molecules and yield insight into the mechanistic basis by which nanoparticles can enhance CNS drug delivery.

## 2. Materials and Methods

### 2.1. Materials

Avidin, dichloromethane (DCM), dimethyl sulfoxide (DMSO), 1,1′-Dioctadecyl-3,3,3′,3′-Tetramethylindotricarbocyanine Iodide (DiR), palmitic acid-NHS, polyvinyl alcohol (PVA), sodium deoxycholate and 10× phosphate buffered saline (PBS) were purchased from Sigma–Aldrich (St. Louis, MO, USA). Ester terminated poly(lactic-*co*-glycolic acid) (50:50 PLGA) was obtained from Lactel (Birmingham, AL, USA). RVG-biotin peptide (sequence: N term-YTIWMPENPRPGTPCDIFTNSRGKRASNG-C2-Biotin) was synthesized by American Peptide Company (Sunnvale, CA, USA).

### 2.2. Nanoparticle Preparation

PLGA nanoparticles were prepared by single-emulsion as previously described [15,20,21]. Briefly, 200 mg of PLGA was dissolved in 2 mL of DCM with 50 μL of DiR (25 mg/mL DiR/ethanol). Avidin-palmitate was prepared by dissolving 25 mg of avidin in 4 mL of buffer (2% *w/v* sodium deoxycholate in 1× PBS). No adjustments to solution pH were made. A volume of 1 mL of palmitic acid (1 mg/mL in buffer) was added drop-wise to the stirring avidin mixture and allowed to react overnight at 37 °C. Avidin-palmitate solution was dialyzed against 1 L of 0.15% sodium deoxycholate in 1× PBS overnight, stirring gently (~160 rpm), and again+ in 4 L of 0.15% sodium deoxycholoate in 1× PBS overnight (Fahmy et al., 2005). The PLGA mixture was emulsified with 2 mL of 5% PVA, 1 mL of avidin-palmitate, and 1 mL distilled H_2_O, and immediately probe sonicated at 40% amplitude for 10 s three times (Fisher Scientific Model 705 Sonic Dismembrator, Waltham, MA, USA). The emulsification was added to 84 mL of 0.3% PVA, and the solvent was allowed to evaporate for three hours while stirring. Particles were washed three times by centrifugation and split evenly for surface modification. To surface modify nanoparticles, 10× molar excess of biotin (ctr-NPs) or RVG-biotin (RVG-NPs) was added with gentle agitation. After incubation at room temperature for 1 h, excess reagent was washed off by centrifugation and trehalose was added for cryoprotection. Nanoparticles were lyophilized and stored at −80 °C.

### 2.3. Nanoparticle Characterization

To assess nanoparticle morphology, representative images of ctr-NPs and RVG-NPS were obtained using scanning electron microscopy (SEM). The average diameter was measured from SEM using ImageJ (v. 1.48, NIH) (*n*>150 measurements per formulation). Hydrodynamic diameter, polydispersity (PD), and zeta potential were assessed by dynamic light scattering (DLS, NanoBrook 90Plus Zeta particle analyzer, BrookHaven Instruments, Hotsville, NY, USA). To measure loading, a solution of nanoparticles (5 mg/mL) in DMSO was prepared. Each solution (40 μL) was added in triplicate to a 96-well plate, and 10 μL of 10% ethanol in DMSO added to each sample well. We have previously reported that PLGA nanoparticles encapsulating DiR by this method are stable in aqueous media with <5% release of DiR after 24 h of incubation [15]. Fluorescence intensity was read on a plate spectrophotometer (Tecan infinite 2000, 750/780 nm excitation/emission) and compared to a standard curve to determine loading. The standard curve was prepared using a 5 mg/mL control nanoparticle solution in DMSO. This control solution (40 μL) was spiked with known DiR concentration dilution (10 μL) prepared from 2.5 mg/mL working solution of DiR in 10% ethanol and 90% DMSO.

### 2.4. Nanoparticle Administration

Female BALB/c mice (6–8 weeks) were purchased from Charles River Laboratories, USA. All experimental procedures were performed in compliance with Barrow Neurological Institute’s Institutional Animal Care and Use Committee (IACUC) regulations (Protocol 444, 02/18/2016.). Mice were housed in a 12:12 light:dark cycle. Food and water were provided ad libitum. We did not control for differences in estrus cycle, acknowledging that this approach could increase biological variability. For intranasal administration, nanoparticles were resuspended in sterile saline, vortexed briefly, and sonicated for 10 min in a bath sonicator. Mice were anesthetized with a ketamine/xylazine cocktail (90/10 mg/kg) and then administered 20 μL of 200 mg/kg of nanoparticles by pipette (5 μL right nostril, 1 min pause, 5 μL left nostril, 5 min pause, ×2). This dosing strategy was developed in a preliminary study. Anesthesia facilitated consistent dose administration by enabling the neck to be maintained at a constant angle. Administrations were provided in alternating nostrils to enable maximum exposure of the administered dose without risk of blocking the airway or producing coughing. We note that higher volumes or alternative dosing paradigms are possible and described by others [22]. At set time intervals (0.5, 2, 6 h) mice were deeply anesthetized with an overdose of ketamine/xylazine. Approximately 200 μL of blood was collected via cardiac puncture and centrifuged at 2000× *g* for 10 min to obtain plasma. Mice were perfused with heparinized saline (10 U/mL) until the liver cleared and decapitated along the atlano-occipital joint. Whole brains were removed and immediately dissected into major anatomical regions, including olfactory bulb, cortex, striatum, midbrain, hippocampus, cerebellum, and brainstem. The spine was cut at the lower lumbar region, and the spinal cord was extracted by applied pressure to the spinal canal opening with a 1ml syringe of distilled H_2_O. Spinal cords were dissected into major anatomical parts (cervical, thoracic, lumbar, and sacral) using the spinal cord intumescences as guides. To isolate the trigeminal nerve, the whole brain was first removed from the cranial cavity. The trigeminal nerve was cut at the sensory root where it enters the brain stem and also separated from the mandibular branch. The ophthalmic and maxillary branches were then followed and cut where the two branches enter the base of the skull. Relevant peripheral organs were extracted and rinsed in distilled H_2_O, including the heart, lungs, kidneys, uterine horns, muscle from hind limbs, and muscle from the spinal region. All tissue was placed in pre-weighed Eppendorf tubes and stored at −80 °C until further processing. Control blood plasma and tissue was also collected from mice that did not receive an injection (*n* = 13). These control samples were used to construct control curves.

### 2.5. Tissue Homogenization

Biodistribution of encapsulated payload was measured as previously described [23]. Peripheral organ tissue was thawed on ice and finely minced into a pulp. The 10% *w/v* distilled H_2_O was added to each sample. Peripheral tissues were physically disrupted in a bead homogenizer (10 min at 10 speed setting) and lysed by probe sonication (40% amplitude for 10 s, 2×) while holding tubes on ice. CNS tissue was thawed on ice and 10% *w/v* distilled H_2_O was added to each sample. Tissue was subjected to probe sonication (40% amplitude for 10 s, 2×) to homogenize. CNS homogenates (40 μL) and peripheral organ homogenates (50 μL) were added to a 96-well plate with 10 μL of DMSO. Fluorescence intensity was read on a plate spectrophotometer (Tecan infinite 2000, 750/780 nm excitation/emission). Tissue homogenates were obtained from control subjects that did not receive nanoparticles, spiked with known quantities of DiR/PLGA to construct a control curve that covered the entire fluorescence range for each tissue type. All samples were read in triplicate, and arbitrary units were converted to ng DiR/g tissue utilizing the linear portion of each control curve.

### 2.6. DiR Imaging

For visualization of DiR delivery to the trigeminal nerve and whole-brain slices, tissues were imaged on a LI-COR Odyssey Clx (LI-COR Biosciences, Lincoln, NE, USA). Brain and trigeminal nerve tissues were placed on a glass slide and scanned using the 700 nm laser channel.

### 2.7. Data Analysis

All data analysis was done on GraphPad Prism 8 software. Statistical significance of differences in peripheral tissue biodistribution was evaluated by two-way ANOVA (*α* = 0.05) followed by post hoc testing at a confidence level of 0.05.

## 3. Results

### 3.1. Nanoparticle Characterization

**Figure 1:**
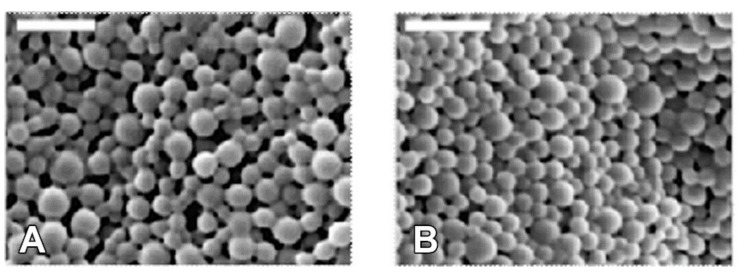
Nanoparticles were spherical and exhibited smooth morphology. (**A**) RVG-NPs. (**B**) Ctr-NPs. Scale bar = 500nm.

Nanoparticles loaded with DiR were formed by single emulsion and surface modified with RVG after fabrication via avidin-biotin interaction. The nanoparticles utilized in these studies were taken from a large pooled batch whose characterization was previously reported and is reiterated here (Table 1 and [15]). Loading was calculated as the average weight percent of the DiR encapsulated in the final yield of PLGA, which resulted in a 0.26% *w*/*w* loading with 38.1% encapsulation efficiency. SEM imaging (Figure 1) demonstrated that nanoparticles were relatively monodisperse, possessing an average diameter of 129 ± 36 nm and 141 nm ± 31 nm for RVG-NPs and ctr-NPs nanoparticles, respectively. Diameters measured by DLS were 188 ± 44 nm and 237 ± 56 nm for RVG-modified and control nanoparticles, respectively. The increase in size for DLS measurements relative to SEM measurements is expected as a result of hydration of the nanoparticle and formation of aggregates. Nanoparticles possessed a near-neutral surface charge of 0.36 ± 1.76 mV and 1.69 ± 0.95 mV for RVG-NPs and ctr-NPs, respectively.

### 3.2. Whole-Organ Biodistribution

**Figure 2:**
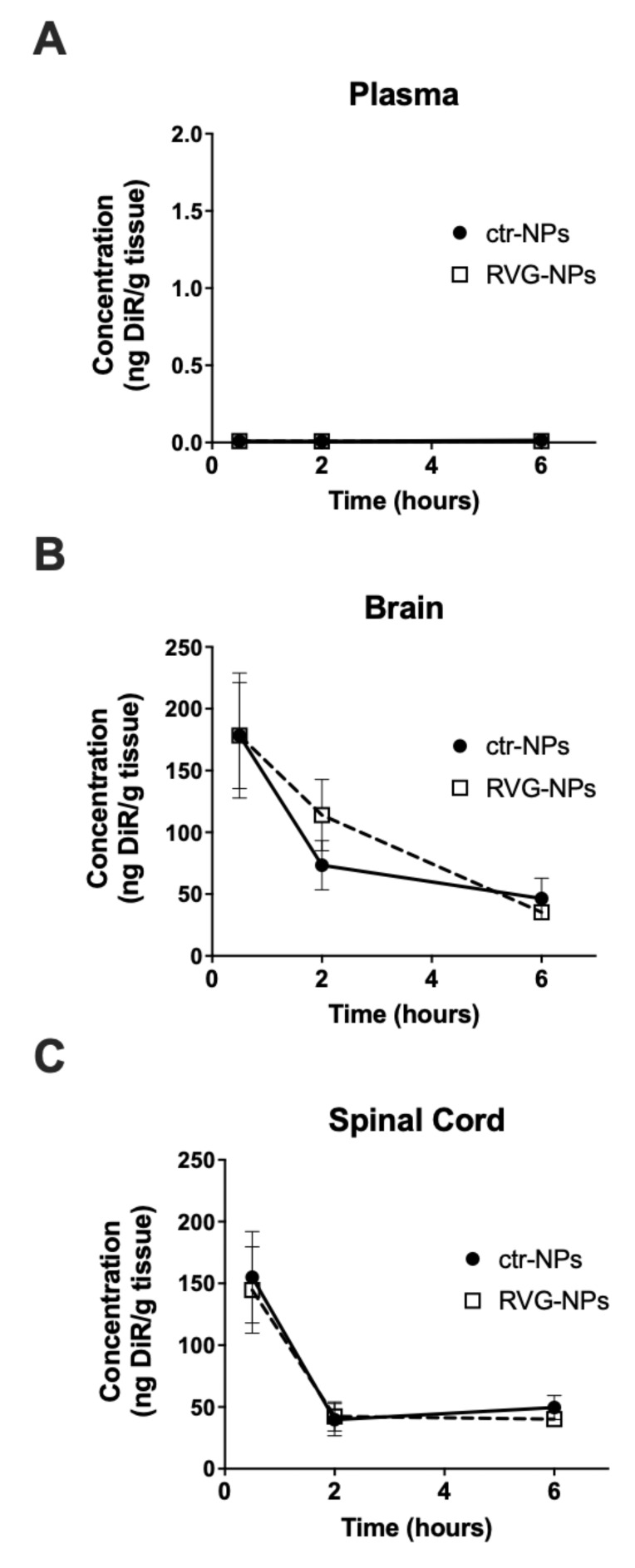
Time-dependent concentration profiles of 1,1′-Dioctadecyl-3,3,3′,3′-Tetramethylindotricarbocyanine Iodide (DiR)-loaded poly(lactic-*co*-glycolic acid) (PLGA) nanoaprticles following intranasal administration. Concentration of DiR in (**A**) blood plasma, (**B**) whole brain, and (**C**) whole spinal cord after intranasal administration. Graphs show the mean ± SEM (*n* = 5–6).

To evaluate delivery of the small molecule DiR, healthy BALB/c mice were administered nanoparticles at a polymer dose of 200 mg/kg. Mice were sacrificed 0.5, 2, and 6 h after administration of nanoparticles. Time-dependent concentration profiles in plasma and CNS tissues are shown in Figure 2. Circulating levels of payload after intranasal administration were below the lower limit of detection, which was 2 ng DiR/mL plasma. The concentration of payload in the brain and spinal cord was initially high and decreased with time. When considering plasma, whole brain, or whole spinal cord, delivery to whole brain tended to be higher for RVG-NPs compared to ctr-NPs 2 h after intranasal administration. However, targeting did not produce a statistically significant increase in delivery to whole brain or whole spinal cord at any time point (*p* > 0.05).

**Figure 3:**
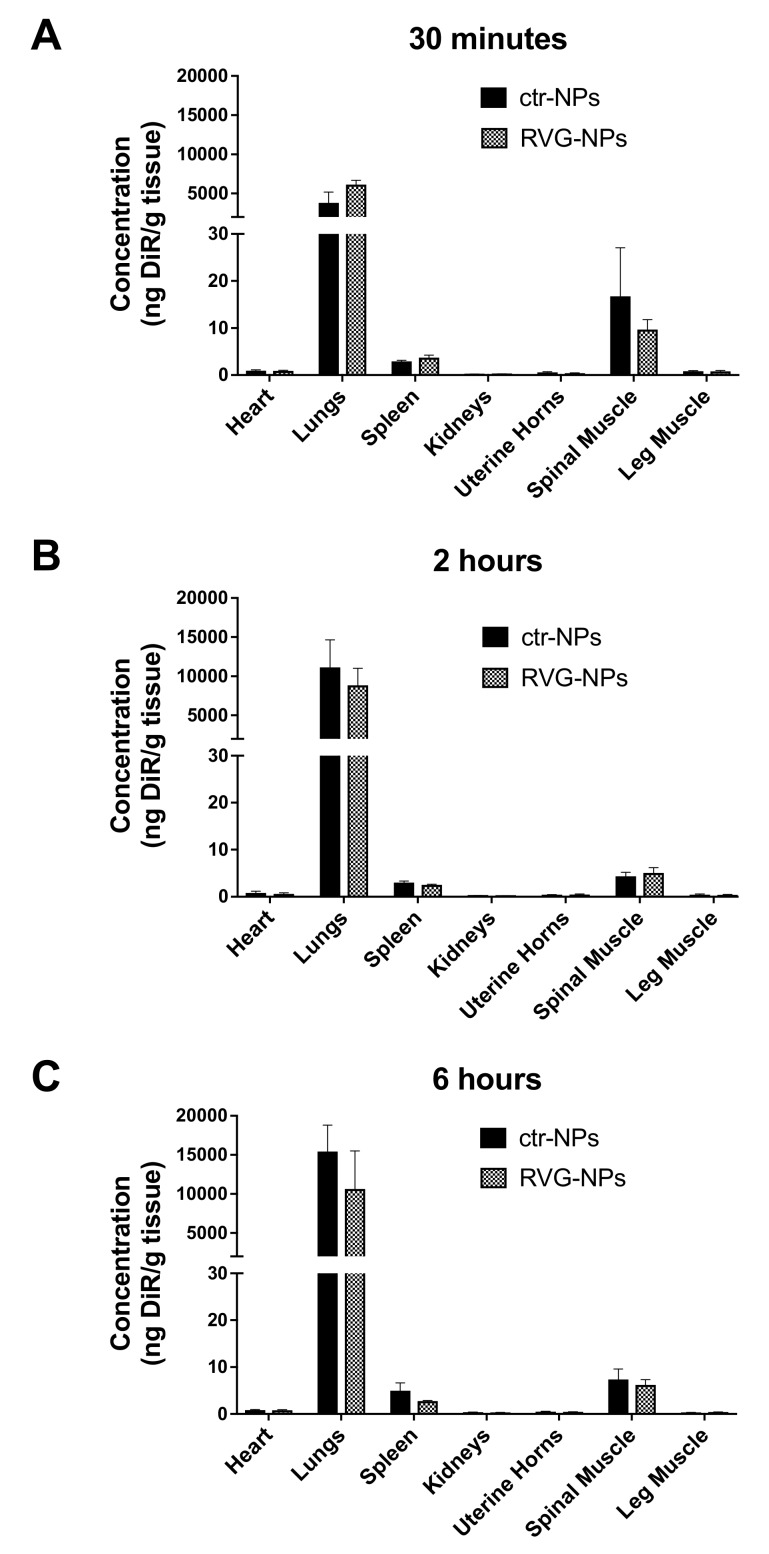
Biodistribution of DiR-loaded PLGA nanoparticles following intranasal administration. DiR concentration was measured in peripheral organs. (**A**) 0.5 h, (**B**) 2 h, (**C**) 6 h. Graphs show the mean ± SEM (*n* = 5–6).

The concentration of payload was also measured in peripheral organs and tissues, including the heart, lungs, spleen, kidneys, uterine horns, spine muscle, and leg muscle (Figure 3). Low concentrations of DiR were measured in all peripheral tissues with the exception of the lungs. A low level of payload was also detected in the spleen and the spinal cord muscle. When considering whole peripheral organs, there was no effect of targeting at any time point (*p* > 0.05).

### 3.3. Spatial Biodistribution Within the CNS

**Figure 4:**
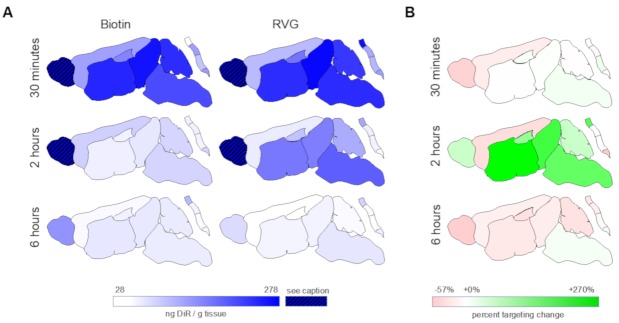
Delivery of payload from intranasally administered nanoparticles varies by central nervous system (CNS) region and surface modification. (**A**) Magnitude of DiR delivered from ctr-NPs (left) or RVG-NPs (right) nanoparticles. Data are scaled to min/max concentration for the entire data set excluding the olfactory bulb. Values for DiR delivery to the olfactory bulb that are out of range for visualization on this scale are listed in Table 2. (**B**) Percent targeting change calculated by dividing the concentration of DiR delivered from RVG-NPs nanoparticles by the concentration of DiR delivered from ctr-NPs nanoparticles for each tissue region. Data are scaled to the min/max concentration for the entire data set such that a value of 0 represents no difference in delivery from a targeted nanoparticle compared to a control nanoparticle.

The spatial distribution of payload within the CNS after intranasal administration of nanoparticles was evaluated by dissecting the brain and spinal cord into its major anatomical regions: olfactory bulb, cortex, striatum, midbrain, hippocampus, cerebellum, brain stem, cervical spinal cord, thoracic spinal cord, lumbar spinal cord, and sacral spinal cord (Figure 4 and Table 2). A two-way ANOVA yielded tissue region as a significant source of variation for all time points. Targeting was a significant source of variation for the 2 h time point only. Delivery was highest in the olfactory bulb compared to other brain regions for all time points evaluated. Payload was detected in regions far caudal to the site of administration within 30 min of nanoparticle administration, including in the brain stem and various segments of the spinal cord. Payload concentration in all brain regions was highest 30 min following nanoparticle administration in all brain regions analyzed, after which it tended to decrease or be roughly maintained. Although targeting did not significantly enhance payload delivery to whole brain or whole spinal cord, examination of the spatial pattern of distribution yielded evidence that payload delivery was much higher in tissue regions that are close to ventral surface of the brain (striatum, midbrain, brainstem,) and much reduced in tissue regions far from the ventral surface of the brain (hippocampus, striatum, cerebellum, thoracic spinal cord, lumbar spinal cord, and sacral spinal cord).

**Figure 5:**
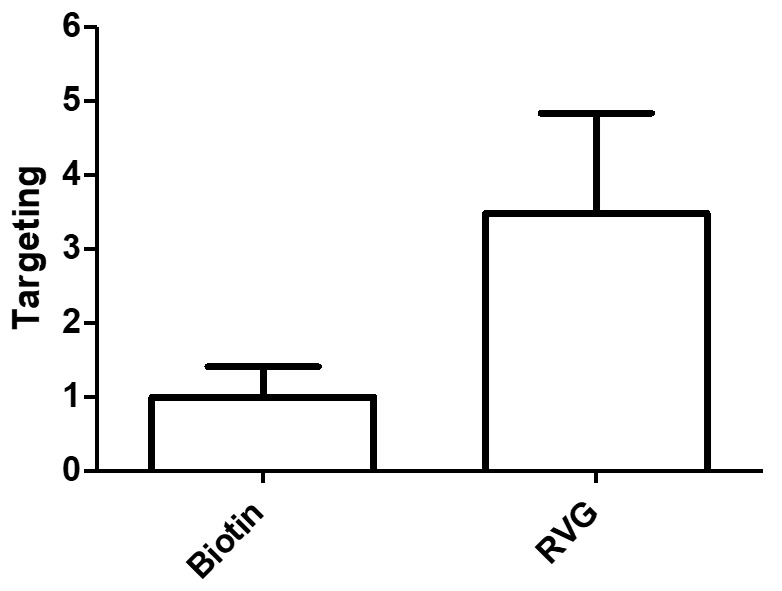
DiR delivery to the trigeminal nerve 2 h after intranasal administration of nanoparticles was much higher for RVG-NPS compared to ctr-NPs (*p* = 0.05). Targeting is defined as the RVG-NP signal divided by the ctr-NP signal (*n* = 5 per group).

**Figure 6:**
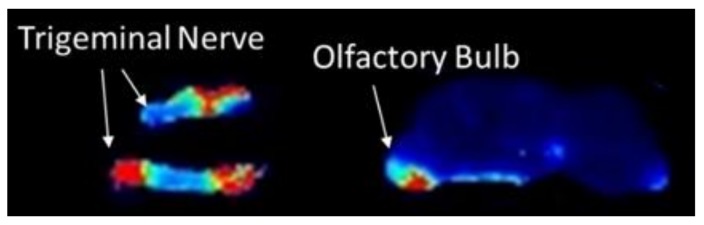
Spatial distribution of DiR in the brain two hours following intranasal administration of nanoparticles. DiR signal is highest in the trigeminal nerve and restricted regions of the ventral aspects of the brain, including striatum, midbrain, and brainstem.

**Figure 7:**
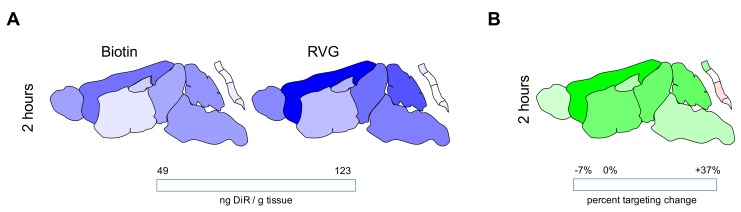
Delivery of payload from intravenously administered nanoparticles varies by CNS region and surface modification; these data were previously published [15] and are replicated here for direct comparison to Figure 5. (**A**) Magnitude of DiR delivered from ctr-NPs (left) or RVG-NPS (right) nanoparticles. Data are scaled to min/max concentration for the entire intravenous data set. (**B**) Percent targeting change calculated by dividing the concentration of DiR delivered from RVG-NPS nanoparticles by the concentration of DiR delivered from ctr-NPs nanoparticles for each tissue region. Data are scaled to the min/max concentration for the entire intravenous data set such that a value of 0 represents no difference in delivery from a targeted nanoparticle compared to a control nanoparticle.

To investigate whether the trigeminal nerve was involved in delivery, we administered nanoparticles intranasally to a separate cohort of mice and removed the trigeminal nerve 2 h post-administration. Payload delivery from targeted nanoparticles to the trigeminal nerve was approximately 3.5× higher than control nanoparticles (*p* = 0.05; Figure 5), which was a higher targeting ratio than what was measured anywhere else in the brain or spinal cord. The pattern of delivery observed for quantitative extraction of DiR from tissue homogenates following intranasal administration matched qualitative assessment of whole-brain slice and trigeminal nerve, where it was evident that signal near to the trigeminal nerve was very high and signal far from the trigeminal nerve was much lower (Figure 6). When the tissues close to the trigeminal nerve were analyzed individually, delivery from RVG-NPs was significantly higher than payload delivery from ctr-NPs (*p* = 0.007, *p* = 0.023, and *p* = 0.041 for striatum, midbrain, and brainstem, respectively). Delivery to the cervical spinal cord tended to be higher for RVG-NPs compared to ctr-NPs, although this difference did not reach statistical significance (*p* = 0.07). These spatial patterns of distribution for both payload magnitude and targeting are in stark contrast to what we previously observed for intravenous administration of the same nanoparticles, where both delivery and targeting were highest along the dorsal surfaces of the brain, particularly the cortex, and lowest along the ventral surfaces of the brain (Figure 7, [15]).

## 4. Discussion

Carrier-based strategies for drug delivery are employed with three major goals: 1) to increase solubility of drugs, 2) to enhance absorption or bioavailability of drugs, and 3) to prolong residence time of drugs or alter distribution within target tissue compartments. Polymeric nanoparticles have attracted considerable attention as drug carriers due to a number of key advantages, particularly their modularity for altering characteristics such as size, shape, and surface features. PLGA is a biodegradable and biocompatible polymer that is often used for drug delivery for a number of well-established, favorable features, including biodegradability, biocompatibility, and the ability to encapsulate a wide variety of molecules for sustained release. PLGA has thus been used extensively to enable or improve drug delivery via both parenteral and intranasal routes [24,25].

The goal of this work was to study spatiotemporal distribution of payload delivery from CNS-targeted nanoparticles. We sought to test the hypothesis that payload exposure to the CNS would be increased following intranasal administration of RVG29-targeted nanoparticles compared to non-targeted nanoparticles. This active targeting approach with RVG29 aims to increase interaction with the underlying nasal epithelium, specifically areas associated with nose-to-brain pathways [25]. Similar targeting approaches have been successfully used to increase CNS delivery in the past with surface modifiers including wheat germ agglutinin [26], arginylglycylaspartic acid (RGD) [27], lactoferrin [28], and the transactivator of transcription peptide (TAT) [29]. Our data demonstrate that while RVG29 modification of PLGA nanoparticles did not enhance whole-brain or whole-spinal cord delivery, significant improvements in delivery were observed within specific regions of the CNS in close spatial proximity to the trigeminal nerve.

Substances delivered to the intranasal cavity can be absorbed into the body by several routes [9]. One possibility is that intranasally administered molecules become absorbed into system circulation through the highly vascularized nasopharyngeal mucosae. By this mechanism, some highly brain penetrant molecules administered intranasally can achieve delivery across the BBB by similar mechanisms as if they had been administered intravenously. Intranasally applied substances can also be inhaled and reach periphery via delivery to the lungs or be cleared to the gastrointestinal tract [9]. It is common to observe high payload delivery to the lungs following intranasal administration [22]. In the present study, DiR concentrations were high in the lungs, which supports inhalation of a portion of the administered dose, although plasma levels of DiR did not exceed the lower limit of detection, which suggests minimal systemic absorption. A small quantity of DiR was measured in the spleen, which likely reflects clearance of nanoparticles by the reticuloendothelial system. Interestingly, DiR was measured at a detectable concentration in muscle near the spinal cord. It is unlikely that this signal originates entirely from circulating DiR, given the low plasma levels and lack of signal in most other peripheral organs. We have recently demonstrated that nanoparticles infused directly into cerebrospinal fluid (intrathecal injection via the cisterna magna) localize with dorsal root ganglia [30], whose exiting nerves would provide a direct pathway to skeletal muscle. Of note, native rabies virus utilizes the opposite path (skeletal muscle to motor neurons) to invade the CNS.

There are two major routes by which nanoparticle or payload could bypass systemic delivery to reach the brain and spinal cord more directly: the olfactory pathway and the trigeminal pathway [9]. By the olfactory route, molecules can move along olfactory neurons that extend from the nasal cavity to the olfactory bulb. This can occur via endocytosis followed by axonal transport, diffusion along lipid membranes, or flow with CSF along the perineural space. Transport along the trigeminal nerve can also involve cellular internalization, axonal transport, or pericellular fluid. For either the olfactory or trigeminal routes, access to the CSF is possible [31,32].

Here, we observed that DiR was delivered across the entire CNS with very rapid kinetics. It is noted that DiR is within a family of carbocyanine dyes that are well documented to stain both fixed and living neuronal tissue by insertion and lateral diffusion along lipid membranes [33]; DiR has been shown to undergo both retrograde and anterograde transport in neurons [33,34]. Appearance of DiR at the cervical spinal cord within thirty minutes of intranasal administration is too rapid to be accounted for by diffusion or axonal transport, emphasizing a role for fluid convection. These kinetics support transport of nanoparticle or released payload along perivascular, perineuronal, or lymphatic spaces [35,36]. The very high levels of DiR in the olfactory bulb and in tissue regions in spatial proximity to the trigeminal nerve suggest that both olfactory and trigeminal pathways play an important role. Appearance of DiR in distal CNS regions and muscle provides evidence of CSF involvement. Thus, we find evidence that delivery to the CNS is achieved by multiple pathways following intranasal administration of nanoparticles.

We observed that DiR is cleared from the CNS relatively rapidly following intranasal administration of nanoparticles. Carbocyanine dyes are regarded to be relatively stable once associated with lipid membranes and have been used to track individual cells for days to months in vitro and in vivo [33,37]. The clearance of DiR that was observed is far more rapid than what would be expected for free molecule embedded within the lipid rich environment of the brain or spinal parenchyma. It is therefore possible that a significant fraction of DiR remains entrapped within the nanoparticle, and that the nanoparticle itself is cleared via mucous or from the parenchyma [21,38].

Toward the long-term goal of improving CNS delivery, nanoparticles were surface modified with the CNS-targeting peptide RVG29. RVG29 has known tropisms for adhesion proteins and receptors associated with the blood brain barrier, the neuromuscular junction, and the terminal ends of olfactory and trigeminal nerves. These targets include include GABA_B_, nicotinic acetylcholine receptors (nAChRs), neural cell adhesion molecule (NCAM), and p75 receptors [39,40]. It has been shown previously that intranasal inoculation of different strains of rabies virus resulted in infection of the brain by penetration through both olfactory and trigeminal pathways [41,42]. Recent data published by Rassu and colleagues [43] specifically explored RVG29 complexed with siRNA for nose-to-brain delivery utilizing cellular assays, although in vivo delivery studies were not performed. We observed here that while whole-brain or whole-spinal cord targeting was not achieved by modifying nanoparticles with RVG29, targeting was robustly observed in tissue regions in close proximity to the trigeminal nerve. These data support the use of RVG29 as an intranasal targeting ligand for treatment of diseases that affect the trigeminal nerve (such as migraine or trigeminal neuralgia), and delivery to the ventral surfaces of the brain or potentially the olfactory bulb and the upper spinal cord remain open possibilities.

An intriguing result of this work arises through comparison of spatial maps produced by intranasal administration to spatial maps produced by intravenous administration. When nanoparticles were administered intravenously, the highest level of payload delivery was consistently observed in the cortex [15]. In this prior work, we identified a strong, direct relationship between cerebral blood volume and DiR delivery across the CNS for intravenously administered nanoparticles. Targeting maps suggested that enhancements in payload delivery from RVG29-modified nanoparticles following intravenous administration most closely related to the spatial distribution of GABA_B_ receptors expressed on endothelial cells of the BBB and BSCB. These previously described intravenous data supported a model for nanoparticle drug delivery by which enhancements in nanoparticle interactions with endothelial cells enabled passive diffusion of payload into the parenchyma. Here, we observed that both payload quantity and targeting achieved by CNS region were fundamentally distinct for intranasally administered nanoparticles compared to intravenously administered nanoparticles. Both delivery and targeting to the cortex were low, while higher delivery and targeting was observed for tissues near to the trigeminal nerve. There was no relationship between cerebral blood volume and delivery for intranasally administered nanoparticles (data not shown). Thus, comparison to intravenous results affirms a role for direct nanoparticle delivery to the brain following intranasal administration that does not rely on re-uptake of molecules from blood to the CNS. Importantly, the spatial maps suggest that utility of the intranasal route will depend heavily on target tissue region.

There are several major avenues for future work identified through these studies. First, it is conceivable that the targeting effects seen were only indirectly provided by RVG29 due to charge and non-specific nanoparticle interactions with the mucus rather than any specificity and affinity to a complimentary receptor. We did not measure rheological properties of the intranasally administered solutions. Although the colloidal suspension is dilute (2.5 *wt*%), RVG29-modified nanoparticles could be more viscous, or more mucoadhesive, which would increase nanoparticle residence time in the nasal cavity via nonspecific means. To directly test the latter hypothesis, it would be necessary to saturate receptors or downregulate their expression within the nasal cavity and relevant structures of the brain. Given the diverse possible targets for RVG29 (at least 4 possible receptors) and their far distance from the site of administration (i.e., farther than free ligand could be expected to diffuse), this is not easily accomplished in vivo. An additional confounding factor is our inability within this experimental context to determine whether the DiR that has been measured in tissue is still retained within the nanoparticle or has been released. This is a significant concern with biodistribution studies that we have directly addressed in prior work [15,44]; future studies would benefit from consideration of the fate of the nanoparticle itself following intranasal administration. This is an especially intriguing avenue for future work given the deposition of payload in muscle that might suggest transport of nanoparticles or payload within the CSF.

There has been some evidence to suggest intact nanocarriers have capacity to transport along an intact trigeminal nerve. In a recent study, Li and colleagues utilized aggregation-caused quenching probes to track the in vivo fate of PCL nanoparticles, and reporting slow transport of intact nanoparticles along the trigeminal nerve to the brain stem, but not along the olfactory nerve [45]. Ahmed and collegaues also detected some presence of 100nm vehicle in the trigeminal nerve, though model cargoes DiR and C6 were ultimately delivered to the brain in free form [46]. The majority of the trigeminal nerve resides outside of the CNS, surrounded by a layer of dura. Rapid appearance of payload in the brainstem could be due to intact nanoparticles moving along this pathway to exit in the brainstem. Taken in sum, our and prior data suggest that it is possible that nanoparticles could transit the trigeminal nerve, with slow release of encapsulate molecules to neighboring brain tissues.

We expect that enhancements in RVG29 targeting via the intranasal route will rely on penetration of nanoparticles through mucous, since the kinetics of delivery are too fast to be accounted for the movement of free dye. We previously demonstrated aqueous instability of the peptide resulted in loss of targeting effects [15]. Given the quick mucosal turnover of intranasally administered particles, we do not suspect this to be a driving factor in lack of targeting at later time points. An increasing body of evidence suggest that nanoparticles are relatively immobile in mucus, and development of mucoadhesive formulations is a more widely studied strategy to improve availability for nose-to-brain delivery by increasing residence time in the nasal cavity [25,47]. In a seemingly counter-intuitive approach of addressing mucillary clearance, increased entanglement of carrier with mucin fibers can increase residence time in the nasal cavity. One new and emerging approach is to develop mucus penetrating nanoparticles with a goal of increasing accumulation on the underlying epithelia. It has been demonstrated that while uncoated PLGA nanoparticles have extremely limited mobility within a mucus environment, the addition of a PEG or Pluronic F-127 outerlayer can improve diffusion by >1000 fold [47]. Therefore, in considering future directions for this work, we suggest that the combination of either PEG coating or mucoadhesive capability with a targeting ligand like RVG29 could significantly improve targeting prospects.

## 5. Conclusions

Intranasal administration of DiR-loaded nanoparticles enables a spatiotemporal pattern of delivery to the CNS that is fundamentally distinct from what has previously been observed for intravenous administration. Our data support a complex scenario in which olfactory, trigeminal, and CSF dissemination all contribute to CNS delivery of nanoparticle encapsulated payload. Delivery of DiR to the CNS following intranasal administration appears to be independent of any systemic pathway. RVG surface modification provides little targeting benefits to the CNS when measured in the whole brain or whole spinal cord, although significant targeting to specific tissues near to the trigeminal nerve was observed. Ultimately, intranasal administration is a promising strategy to effectively deliver therapeutics directly to the CNS while minimizing systemic absorption and peripheral exposure. Exploring the therapeutic efficacy of intranasally administered hydrophobic small molecule drugs for the treatment of CNS disease will be a future direction of this work.

## Figures and Tables

**Table 1:** Characteristics of Rabies Virus Glycoprotein modified, targeted nanoparticles (RVG-NPs) and control, non-targeted nanoparticles (Ctr-NPs) used in biodistribution studies [15].

Name	SEM				DLS		
	Loading (%)	EE^1^ (%)	Size (nm)	PD^2^ (nm)	Size (nm)	PD (nm)	Zeta Potential (mV)
RVG-NPs	0.26	38.1	129	36	188	44	0.36 ± 1.76
NPs	-	-	141	31	238	56	1.69 ± 0.95

^1^ EE = encapsulation efficiency; ^2^ PD = polydispersity.

**Table 2:** Concentration of DiR (ng/g tissue) measured within specific CNS regions of the central nervous system (CNS) following intranasal administration of nanoparticles.

Time	CNS Region	Ctr-NPs^1^	RVG-NPs^1^	Targeting^2^
0.5 h	Brain			
	Olfactory Bulb	627.8 ± 507.6	325.7 ± 189.8	−48%
	Cortex	120.7 ± 95.5	95.5 ± 93.3	−21%
	Striatum	241.8 ±130.9	234.4 ± 217.1	−3%
	Midbrain	260.3 ± 98.4	271.2 ± 135.6	+4%
	Hippocampus	148.5 ± 115.6	164.3 ± 127.7	+11%
	Cerebellum	234.4 ± 78.6	223.8 ± 128.1	−5%
	Brain Stem	205.6 ± 89.5	235.7 ± 174.6	+15%
	Spinal Cord^3^			
	SC-C	278.1 ± 98.6	263.7 ± 155.3	−5%
	SC-T	122.1 ± 76.4	111.8 ± 77.2	−8%
	SC-L	85.2 ± 72.4	99.2 ± 74.4	+16%
	SC-S	126.8 ± 107.3	121.7 ± 87.7	−4%
2 h	Brain			
	Olfactory Bulb	227.8 ± 257.1	359.9 ± 244.4	+58%
	Cortex	73.7 ± 101.4	46.3 ± 25.9	−37%
	Striatum*	43.4 ± 19.4	160.4 ± 93.6	+270%
	Midbrain*	51.2 ± 20.1	154.7 ± 108.8	+202%
	Hippocampus	41.5 ± 21.9	87.4 ± 83.6	+111%
	Cerebellum	68.4 ± 79.6	108.9 ± 84.5	+59%
	Brain Stem*	70.0 ± 40.8	186.1 ± 139.7	+166%
	Spinal Cord			
	SC-C	40.1 ± 35.7	114.3 ± 106.3	+185%
	SC-T	40.7 ± 20.4	42.5 ± 12.6	+5%
	SC-L	38.2 ± 29.1	39.0 ± 3.7	+2%
	SC-S	69.2 ± 107.6	29.8 ± 10.0	−57%
6 h	Brain			
	Olfactory Bulb	133.5 ± 222.6	63.0 ± 27.7	−53%
	Cortex	33.8 ± 26.9	27.6 ± 10.5	−18%
	Striatum	51.6 ± 40.0	38.1 ± 10.5	−26%
	Midbrain	50.6 ± 37.2	39.3 ± 27.8	−22%
	Hippocampus	41.1 ± 35.3	28.2 ± 9.6	−31%
	Cerebellum	47.3 ± 37.8	32.0 ± 11.4	−32%
	Brain Stem	46.2 ± 26.3	51.8 ± 29.7	+12%
	Spinal Cord			
	SC-C	96.2 ± 74.1	70.7 ± 31.8	−26%
	SC-T	37.6 ± 9.1	31.0 ± 10.2	−18%
	SC-L	30.2 ± 17.4	27.8 ± 9.4	−8%
	SC-S	37.9 ± 8.8	43.3 ± 22.5	+14%

^1^ Mean ± standard deviation for control, non-targeted nanoparticles (ctr-NPs) and Rabies Virus Glycoprotein, targeted nanoparticles (RVG-NPs); *n* = 5–6 mice per group. ^2^ Targeting is defined as the percent increase or decrease in RVG relative to biotin; ^3^ SC = spinal cord, C = cervical, T = thoracic, L = lumbar, and S = sacral. * Delivery from RVG-NPs was statistically significantly greater than delivery from ctr-NPs.
